# Exploring the potential impact of applying web-based training program on nurses’ knowledge, skills, and attitudes regarding evidence-based practice: A quasi-experimental study

**DOI:** 10.1371/journal.pone.0297071

**Published:** 2024-02-08

**Authors:** Rasha A. Mohamed, Muhanad Alhujaily, Faransa A. Ahmed, Wael G. Nouh, Abeer A. Almowafy

**Affiliations:** 1 Department of Nursing, College of Applied Medical Sciences, University of Bisha, Bisha, Kingdom of Saudi Arabia; 2 Department of Medical Laboratory Sciences, College of Applied Medical Sciences, University of Bisha, Bisha, Kingdom of Saudi Arabia; 3 Department of Nursing, College of Applied Medical Sciences in Alnamas, University of Bisha, Bisha, Kingdom of Saudi Arabia; 4 International Islamic Center for Population Studies and Research, Al-Azhar University, Cairo, Egypt; University of Hafr Al-Batin, SAUDI ARABIA

## Abstract

Evidence-based practice (EBP) has gained significant importance in clinical practice worldwide, including in nursing. This study aimed to explore the potential impact of applying a web-based training program on nurses’ knowledge, skills, and attitudes regarding EBP. A quasi-experimental pretest-posttest research design was utilized with a purposive sample of 64 professional nurses who agreed to participate. The study took place in different hospitals and primary healthcare centers in the Bisha Governorate, Aseer region, Saudi Arabia. A four-week standardized web-based training program was implemented using an online learning approach. Nurses were provided with an online self-rated data collection tool through the Google Forms platform. The findings indicated a highly significant difference in the total knowledge and EBP skills mean scores of the post-intervention (53.08±15.9) and (66.03±8.95), respectively compared to pre-intervention (P<0.05). Additionally, there was marked improvement in the mean scores of the positive attitude of the training sessions post-intervention compared to pre-intervention. The program was also well-received by the nurses in terms of quality and usability. The program has the potential to enhance nurses’ knowledge, skills, and attitudes toward EBP. Therefore, healthcare organizations may consider adopting web-based training as a means of continuing professional education to promote EBP competencies among nurses.

## Introduction

Globally, evidence-based practice (EBP) is considered the gold standard for providing safe, qualified, and cost-effective healthcare [1]. EBP is the process of reviewing, analyzing, and translating the latest scientific evidence into nursing practice to make informed patient care decisions [2]. The use of EBP is strongly correlated with better patient outcomes, including shorter hospital stays, lower readmission rates, lower mortality [3], lower health disparities, and decreased trial and error due to ineffective medical procedures [4]. Moreover, it aids in the standardization of nursing practices, and the development of nurses’ bodies of knowledge [5, 6].

EBP and quality improvement (QI) should be administered by all healthcare workers, notably nurses who act as front-line healthcare providers [7]. However, many nurses reported having gaps in their understanding of how to perform the scientific research required to apply EBP; they did not use EBP regularly or were not dedicated to doing so [1, 6, 8–11].

The Kingdom of Saudi Arabia (KSA) advocates improving nurses’ evidence-based competencies and concentrates on developing a modern, trustworthy healthcare system that provides services with a quality guarantee [12]. However, the adoption of EBP is challenging for nurses in KSA and other nearby nations, including Jordan, Oman, and Iran, owing to a lack of support and implementation hurdles for EBP [13]. Therefore, the implementation of EBP and training on identified barriers is mandatory and should be considered by policymakers [14]. Online education, especially during vocational training, is one of the major ways to increase learners’ EBP competency and, as a result, promote EBP in service organizations [15, 16]. It is effective in improving the accessibility and comprehension of educational content [17, 18].

To boost nurses’ competency in their practices and significantly improve healthcare quality in hospital settings, it is crucial to evaluate their current level of knowledge, skills, and attitudes toward EBP and QI [7, 19, 20]. Moreover, little is known about the nurses’ EBP competencies [21]. Given what has already been said, the current study aimed to explore the potential effect of a web-based training program on nurses’ knowledge, skills, and attitudes regarding EBP. We hypothesized that nurses who had received the web-based training program would have improved their knowledge, skills, and attitudes regarding EBP (primary outcome) and would accept the intervention (secondary outcome).

## Materials and method

### Ethics approval and consent to participate

The researchers conducted the study in accordance with the rules and ethical principles established by the institutional review board of the University of Bisha (UBCOM-RELOC H-06-BH-087/ (0301.23). The study adhered to the ethical standards outlined in the Helsinki Declaration of 1964 and its subsequent amendments. The study took place from November 23, 2022, to April 15, 2023. All participants provided informed consent by completing an electronic form. Additionally, participants were informed of their right to withdraw from the study at any time without having to provide a reason.

### Study design

This study utilized a quasi-experimental, pretest-posttest, and one-group research design. This type of design is an empirical study that aims to estimate the effect of an intervention in a specific population without random assignment [22].

### Participants

Participants were invited to take part in the study through convenience sampling. They were recruited from King Abdallah Hospital, Maternity and Children’s Hospital, Convalescent and Long-Term Care Hospital, as well as primary healthcare centers in Bisha Governorate, Aseer region, Saudi Arabia (Bisha, Nemran, Al Mattar, and Ganoub El Madina). The inclusion criteria were as follows: 1) professional nurses currently employed in various departments at the selected settings for at least one year; 2) holding a bachelor’s degree in nursing sciences or higher; and 3) willing to participate in the study and having signed the informed consent form. Nurses with less than one year of experience and interns were excluded. The required sample size to achieve the study’s aim was determined using the G*Power Program® Version 3.1.9.4. Assuming an α level of 0.05, a β level of 0.20, and a desired power of 80%, with an assumed mean difference of 1.5 before and after the intervention, and an effect size of 1 [23], the sample size was calculated to be 64 nurses.

### The study implementation process

**Fig 1:**
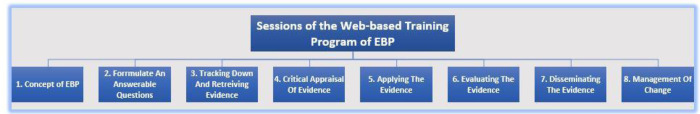
EBP sessions.

Initial data were gathered before the intervention to develop the content of the EBP training program. Researchers extracted scientific content from reliable sources [24, 25]. It is important to note that the nurses were from different nationalities, so the text was written in English. The development of the EBP website was carried out by selecting an educational platform. The link to the website (https://evidence-gate.com/) was distributed to a panel of external reviewers (n = 5) to test the content validity, clarity, consistency, accuracy, applicability, and format of the program. At the same time, the face validity of the training program was assessed by a sample group of nurses (n = 7) who had no experience with EBP. These nurses were given two sessions per week and provided specific feedback to the content developer. A breakdown of the EBP training program sessions is shown in Fig 1. These sessions can be accessed on laptops, computers, and mobile devices.

The web-based intervention was designed as an asynchronous education. An informational session was held on the Blackboard platform affiliated with the University of Bisha to instruct nurses on how to use the learning management system. Each session lasted 45–60 minutes, and the nurses were advised to complete at least two sessions per week. Therefore, the program was intended to be completed in approximately four weeks. Post-learning discussions and comments were designed in the web contact section to gather the nurses’ impressions of the learning experience. The intervention was evaluated using a pre-intervention data collection tool. In the current study, all participants started and completed the intervention, and no participants were excluded from the analysis.

### Instruments

The data collection tool was uploaded electronically to the Google platform. Permission to use the tool was obtained from the copyright holders. The reliability of the tool was tested using Cronbach’s alpha. The results were 0.80, 0.82 and 0.81 for knowledge, skills, and attitudes, respectively indicating a well-accepted reliability. The tool consists of five sections:

I. Personal and occupational data included age, gender, educational level, workplace, years of work experience, and whether they had received any training programs related to EBP and research (Yes/No).

II. The EBP Knowledge Test consisted of 84 questions to assess the participants’ self-perceived knowledge of EBP, both before and after the intervention. The test was adapted from Samy et al. [26] and had been previously validated. It covered topics such as the definition, benefits, and steps of EBP. Participants received a score of (1) for a correct answer and (0) for an incorrect answer. The scores were then averaged to obtain the arithmetic mean and standard deviation. The lowest possible score was zero, and the highest score was 84. A higher score indicated greater knowledge.

III. The EBP Self-reported Skills Scale was used to assess the participants’ self-perceived skills in EBP, specifically their ability to perform each step on their own, both before and after the intervention. The scale was adapted from Samy et al. [26] and had been previously validated. Participants rated their skills on a three-point Likert scale (competent, improving, and incompetent) based on predetermined criteria [30]. Each item was assigned a score: three points for competent, two points for improving, and one point for incompetent. The total number of skills was 27. Therefore, the lowest possible score was one, and the highest score was 81. A higher score indicated greater skill.

IV. The EBP Attitude Scale was used to measure the participants’ attitudes towards EBP, both before and after the intervention. Participants rated their agreement or disagreement with statements on a five-point Likert scale ranging from 1 (strongly disagree) to 5 (strongly agree). The scale was adapted from Samy et al. [26] and had been previously validated. There was a total of 20 attitudes, with a maximum score of 100. The total attitude scores were calculated and categorized into three levels: a highly positive attitude (more than 75%), a positive attitude (75–50%), and a negative attitude (less than 50%). A higher score indicated a more positive attitude [27].

V. The Feedback Regarding the Web-Based Intervention Scale was adapted from El-Refaay et al. [28] and had been previously validated. It consisted of 26 criteria divided into six domains. Participants rated each criterion on a four-point Likert scale ranging from one to four, and they also had the option to provide qualitative comments for criteria requiring a narrative response. The participants’ responses were summarized using arithmetic means and standard deviations.

### Data analysis

Data were entered and analyzed using the Statistical Package for Social Sciences (SPSS) software version 24 (Armonk, NY, USA). The normality of the data was assessed using the Kolmogorov-Smirnov test and presented through descriptive statistics in the form of frequencies and percentages. The arithmetic mean and standard deviation were used for continuous variables and percentages were used for categorical variables. Paired-sample t-test and ANCOVA were used. The effect of the intervention was demonstrated by comparing the participants’ knowledge, skills, and attitudes before and after intervention. An additional relationship was investigated to examine relevant sociodemographic variables. Statistical significance was set at p<0.05.

## Results

### Participant characteristics

Table 1 summarizes the personal and occupational characteristics of the nurses. According to the results, there was a highly significant difference in the total mean knowledge scores between the post-intervention and pre-intervention (*P*≤0.05). Additionally, there was a highly significant difference between the total mean scores of the EBP skills pre-intervention and the mean scores of the post-intervention (*P*≤0.05) (Table 2).

**Table 1:** Distribution of the studied nurses according to their personal and occupational characteristics.

Items	N = (64) (%)
**Age in years** ($\bar{X}$**±S.D)**	35.81±7.27
<25	2(3.1)
25-<35	29(45.3)
35-<45	25(39.1)
>45	8(12.5)
**Sex**	
Male	24(37.5)
Female	40(62.5)
**Educational level**	
Bachelor’s degree (Nursing BSc)	30(46.9)
Post-graduate degree (MSc, Ph.D.)	10(15.6)
Others (Diploma)	24(37.5)
**Workplace**	
Hospitals	52(81.2)
Healthcare centers	12(18.8)
**Years of experience**	13.08±8.19
<5	10(15.6)
5<10	11(17.2)
10<15	18(28.1)
15–20	25(39.1)
**Receiving educational training program related to EBP, and research**	
No	43(67.2)
Yes	21(32.8)

**Table 2:** Comparison of the mean score of knowledge and skills pre- and post-intervention of the web-based training program of EBP.

EBP Knowledge and Skills Domains	Test time n = (64)		Paired t-test	*P*-value	Effect size measures (Cohen’s d)
	Pre-intervention	Post-intervention			
	$\bar{X}$±S.D	$\bar{X}$±S.D			
**Knowledge Domains**					
The concept and benefits of evidence-based practice (8 marks)	0.93±0.61	5.85±2.87	14.88	0.000*	2.37
Asking answerable questions (27 marks)	4.66±3.74	17.84± 5.33	25.00	0.000*	2.86
Acquiring research (18 marks)	2.84±2.54	8.40±4.91	11.22	0.000*	1.42
Critical appraisal (15 marks)	1.32±1.91	11.35±8.15	29.80	0.000*	1.69
Applying, evaluating, and disseminating evidence (16 marks)	1.64± 1.66	9.64±4.38	15.30	0.000*	2.41
**Total knowledge score (84 marks)**	11.47±8.17	53.08± 15.9	28.91	0.000*	3.29
**Skills Domains**					
Asking answerable questions (27 marks) 1–9	9.29±0.06	22.43±3.23	13.73	0.000*	5.75
Acquiring research (24 marks) 10–17	8.45±0.66	19.64±2.92	29.05	0.000*	5.28
Appraising the evidence (21 marks) 18–24	7.50±0.75	16.75±3.05	23.78	0.000*	4.16
Summarizing and synthesizing evidence (9 marks) 25–27	3.15±0.36	7.20±1.14	26.66	0.000*	4.79
**Total skills score (81 marks)**	28.40±1.29	66.03±8.95	33.45	0.000*	5.88

**t**: paired sample t-test ***P***: Significance.

* Significant (p≤0.05).

**Fig 2:**
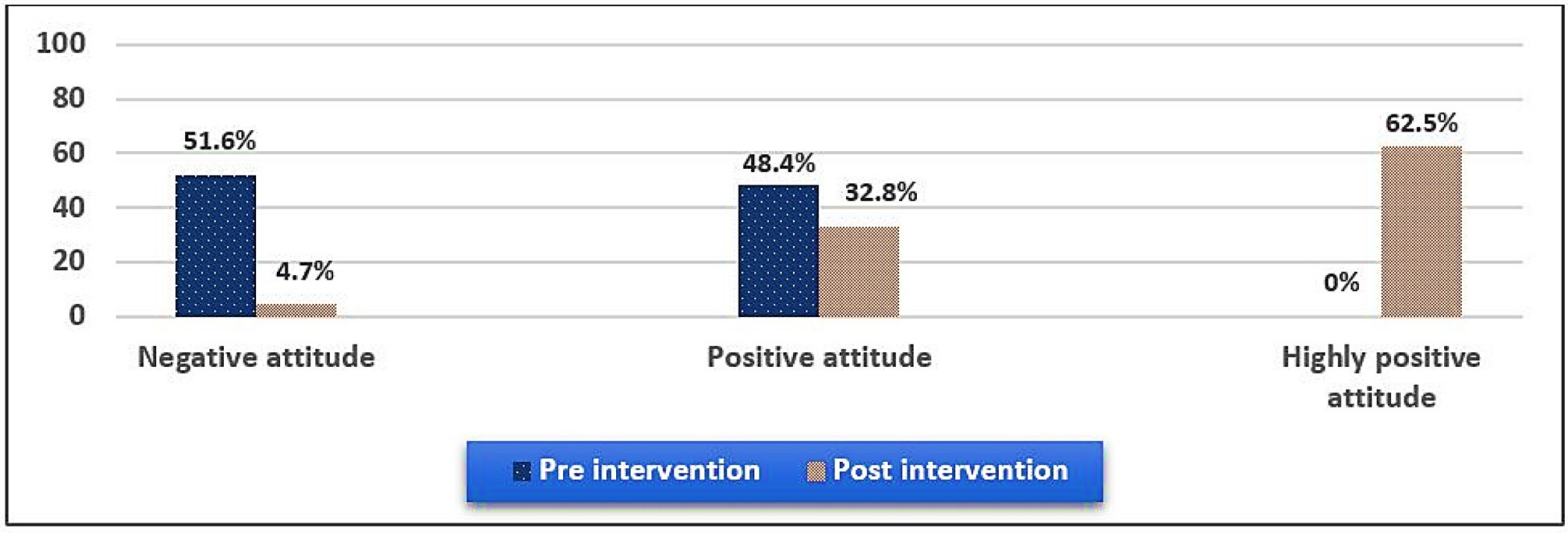
Distribution of the attitude toward EBP pre- and post-intervention.

Fig 2 presents the distribution of the studied nurses according to their attitudes toward EBP pre- and post-intervention.

A significant relationship was found in the mean score of knowledge of the nurses and their workplace, and years of experience. However, no relationship was found between the mean score of the nurses’ knowledge and their age, sex, or academic qualification (Table 3).

**Table 3:** Comparison of the demographic and occupational data of the nurses and their total knowledge score.

Items	Test time n = (64)	
	Pre–Intervention	Post–Intervention
	$\bar{X}$±S.D	$\bar{X}$±S.D
**Age in years**		
<25	15.00±19.79	45.00±29.69
25<35	10.24±7.93	51.48±15.48
35<45	13.45±8.28	58.24±15.81
>45	9.12±5.30	44.12±10.86
**ANCOVA [F, P]**	F = 2.31, P = 0.085	
**Sex**		
Male	13.00±10.06	54.08±16.03
Female	10.53±6.72	52.37±15.98
**t-test [t, P]**	t = 0.55, P = 0.460	
**Academic qualification**		
Bachelor’s degree (Nursing BSc)	14.80±8.64	58.23±15.33
Post-graduate degree (MSc, Ph.D.)	12.22±6.90	57.60±15.21
Others (Diploma)	7.04±5.82	44.58±13.66
**ANCOVA [F, P]**	F = 1.51, P = 0.227	
**Workplace**		
Hospitals	17.25±8.08	65.75±7.36
Healthcare centers	10.11±7.65	27.41±24.42
**t-test [t, P]**	t = 7.55, P = 0.008*	
**Years of experience**		
<5	10.30±8.60	47.40±14.46
5<10	9.00±4.79	47.90±12.11
10<15	10.58±9.27	52.16±18.07
15–20	14.36±10.26	58.00±16.42
**ANCOVA [F, P]**	F = 0.64, P = 0.049	

**F**: Repeated measures ANCOVA **t:** t-test ***P*:** Significance

* Significant (p≤0.05)

A significant relationship was shown between the mean score of the nurses’ EBP skills and their age, workplace, and years of experience. However, no relationship was found between the mean EBP skills score of the nurses and their sex (Table 4).

**Table 4:** Comparison of the demographic and occupational data of the nurses and the total skill score.

Items	Test time n = (64)	
	Pre–Intervention	Post–Intervention
	$\bar{X}$±S.D	$\bar{X}$±S.D
**Age in years**		
<25	28.50±19.79	60.04±1.02
25<35	28.31±1.16	76.10±17.28
35<45	28.40±1.90	81.84±15.29
>45	28.75±1.98	71.12±21.85
**ANCOVA [F, P]**	F = 1.72, P = 0.027	
**Sex**		
Male	28.66±1.37	77.37±19.39
Female	28.25±1.23	77.12±16.10
**t-test [t, P]**	t = 1.19, P = 0.278	
**Academic qualification**		
Bachelor’s degree (Nursing BSc)	28.20±1.21	79.76±15.35
Post-graduate degree (MSc, Ph.D.)	29.30±1.82	88.90±7.82
Others (Diploma)	28.29±0.00	69.16±18.95
**ANCOVA [F, P]**	F = 1.85, P = 0.166	
**Workplace**		
Hospitals	28.32±1.26	74.78±17.80
Healthcare centers	28.75±1.42	87.75±9.31
**t-test [t, P]**	t = 3.49, P = 0.046	
**Years of experience**		
<5	28.60±1.50	68.90±13.09
5<10	28.09±0.83	77.81±20.01
10<15	28.11±1.07	77.88±14.26
15–20	28.54±1.86	81.09±18.72
**ANCOVA [F, P]**	F = 2.05, P = 0.042	

**F**: Repeated measures ANCOVA **t:** t-test ***P*:** Significance

* Significant (p≤ 0.05)

Nurses’ feedback regarding the web-based EBP training program revealed that more than half of them agreed that the course information was meaningful, practicable, and accessible (S1 Table).

## Discussion

The main result of this study indicates that significant improvements in knowledge, skills, and attitudes toward EBP were found among nurses after participating in the web-based training program. Additionally, the program was well accepted by nurses and was found to be useful, flexible, and convenient. One possible explanation for these findings is that the program consistently focused on its objectives throughout all sessions, provided personalized tutoring, utilized structured learning strategies and materials (such as critical evaluation checklists), integrated activities with clinical practice, and offered discussion and feedback. The website was also user-friendly. Similar outcomes have been reported by Ibrahim et al. [29], and our findings are supported by several other studies [30–35]. In line with our study, [36] who found that computer-based education is an effective approach that nursing leaders can use to educate and engage nurses in EBP initiatives and research utilization. In contrast, other studies [37, 38] have reported different results.

Regarding knowledge of EBP, the nurses had limited awareness of EBP prior to the intervention. This could be attributed to the lack of educational programs on EBP for nurses. However, nurses who participated in the web-based training program showed a significant improvement in their EBP knowledge. These findings are consistent with a study by [39], which showed that a brief web-based educational intervention on systematic reviews can enhance healthcare workers’ knowledge temporarily. Like the present study, [40–43]. Moreover, the current study found that nurses who took part in the web-based training program demonstrated improved EBP skills. This could be attributed to their increased awareness of EBP after the intervention, as reported in other studies [40–42, 44, 45].

In terms of nurses’ attitudes towards EBP, the web-based training program had a positive impact, as evidenced by an increase in positive attitude scores after the intervention. This finding aligns with a study that showed a statistically significant improvement in nurses’ perceptions of EBP [46]. Additionally, our study’s results are consistent with those of Chao et al. [41].

Understanding the needs, perspectives, views, and opinions of healthcare professionals (HCPs) regarding the module is essential for developing educational content that addresses their perceived needs and enhances their competencies in implementing EBP [20]. According to our findings, the participating nurses expressed acceptance of the web-based training program and found it useful, flexible, and effective in enhancing their professional skills. Most participants agreed that the program was easy to navigate and delivered at a logical and manageable pace. Similar results were found in a study conducted in Egypt, which concluded that HCPs were receptive to web-based delivery of intervention content [28]. Our study’s findings are consistent with existing literature [17, 40, 44, 47–49], further supporting the acceptability and effectiveness of the training programs in improving nurses’ competencies.

## Conclusion

The results indicated that the web-based training program has the potential to enhance nurses’ knowledge, skills, and attitudes toward EBP. Implementing follow-up interventions, such as journal clubs, may also aid in retaining and improving nurses’ EBP knowledge and skills. However, it is important to acknowledge that web-based health education has its limitations, including the time and effort required by instructors to build and develop the program, teach necessary skills, maintain, and upgrade the platform, and address any technical issues that may arise. Despite these drawbacks, the overall benefits of web-based training outweigh these challenges.

## Recommendations

We suggest that healthcare organizations adopt web-based training as a method for continuing professional education to enhance the competencies required for implementing EBP among nurses. It is also essential to establish the organizational infrastructure and mentoring necessary to support EBP and research utilization. Furthermore, creative strategies are needed to promote a culture of clinical inquiry and motivate nurses to utilize available EBP resources.

## Strengths and limitations

To the best of our knowledge, this is the first study conducted in Saudi Arabia with the aim of improving the utility of a remotely delivered web-based educational intervention, specifically targeting nurses to increase their EBP’ knowledge, skills, and attitudes. The strength of this study lies in the conception of a novel idea and the use of different questions on both the pre- and post-test, which improved the validity of our study.

However, there are several limitations that need to be considered when interpreting the findings. Firstly, the study’s non-probability convenience sample raises the possibility of bias. Secondly, assessing skills based on self-reported responses from participants may lead to overreporting or information bias, which reduces our confidence in the strength of the intervention effect. Lastly, the quasi-experimental design used in this study does not allow us to determine causality without a control group. Therefore, our findings require further validation through controlled experiments with proper control groups. To overcome these limitations, the authors of this study attempted to minimize bias by having a non-biased scorer consistently score the pre-test and post-test. This scorer was blinded to the participants and was not involved in designing the intervention sessions. Additionally, the time interval between the pre-test and post-test was kept short. To generalize our findings, further research is needed on a larger group of nurses, including a control group with similar educational backgrounds.

## Supporting information

S1 Table Distribution of the nurses’ feedback regarding the developed web-based training program of EBP. (DOCX)
