# Correction: Exploring the potential impact of applying web-based training program on nurses’ knowledge, skills, and attitudes regarding evidence-based practice: A quasi-experimental study

**DOI:** 10.1371/journal.pone.0299864

**Published:** 2024-02-27

**Authors:** Rasha A. Mohamed, Muhanad Alhujaily, Faransa A. Ahmed, Wael G. Nouh, Abeer A. Almowafy

There are errors in the Funding statement. The correct Funding statement is: The University of Bisha through the general research project under grant number (UB-GRP-19-1444) funded the study. The funders had no role in study design, data collection and analysis, decision to publish, or preparation of the manuscript.

The Acknowledgements section is missing from the article. The correct Acknowledgements statement is as follows: The authors extend their appreciation to the deanship of scientific research at the University of Bisha for funding this research through the general research project under grant number (UB-GRP-19-1444). Also, we acknowledge the outstanding efforts of the nurses who took part in the study for their assistance and enthusiastic cooperation during the study period.
