# Supplementary material for: Exploring the potential impact of applying web-based training program on nurses’ knowledge, skills, and attitudes regarding evidence-based practice: A quasi-experimental study
Source: PLoS One. 2024 Feb 8;19(2):e0297071. doi: 10.1371/journal.pone.0297071 (PMC10852226; doi:10.1371/journal.pone.0297071)
Supplement: S1 Table — (DOCX) [file pone.0297071.s001.docx]

**Table S1. Distribution of the Nurses' Feedback Regarding the Developed Web-Based Training Program of EBP.**

| **N=64** | | | | | | **Items** |
| --- | --- | --- | --- | --- | --- | --- |
| **Disagree** | | **Agree** | | **Strongly agree** | |  |
| **%** | **N** | **%** | **N** | **%** | **N** |  |
| **The usefulness of the program information (48 marks) ±S.D (40.12±4.6)** | | | | | | |
| 23.4 | 15 | 53.1 | 34 | 23.4 | 15 | Understanding of evidence-based practice |
| 0 | 0 | 68.8 | 44 | 31.3 | 20 | Improve the quality of patient care |
| 0 | 0 | 45.3 | 29 | 54.7 | 35 | Improve practices of healthcare professionals |
| 0 | 0 | 62.5 | 40 | 37.5 | 24 | Acquire useful information |
| 1.6 | 1 | 60.9 | 39 | 37.5 | 24 | Use in the workplace |
| 0 | 0 | 65.6 | 42 | 34.4 | 22 | It is helpful in my work |
| 4.7 | 3 | 59.4 | 38 | 35.9 | 23 | It is meaningful |
| 0 | 0 | 57.8 | 37 | 42.2 | 27 | It is understandable |
| 0 | 0 | 62.5 | 40 | 37.5 | 24 | It is practical |
| 0 | 0 | 57.8 | 37 | 42.2 | 27 | It is satisfactory |
| 9.4 | 6 | 57.8 | 37 | 32.8 | 21 | It is a convenient way for continuing education |
| 0 | 0 | 65.6 | 42 | 34.4 | 22 | Recommend this program to others |
| **Information structure and design activities (44 marks) ±S.D (37.23±4.3**) | | | | | | |
| 0 | 0 | 60.9 | 39 | 39.1 | 25 | Learning objectives are clearly stated in each session |
| 0 | 0 | 40.6 | 26 | 59.4 | 38 | The scope of the sessions is clearly stated |
| 0 | 0 | 54.7 | 35 | 45.3 | 29 | The content of the sessions was clearly written |
| 0 | 0 | 60.9 | 39 | 39.1 | 25 | The content of the sessions was accurate |
| 1.6 | 1 | 65.6 | 42 | 32.8 | 21 | The content of the sessions was easy to understand |
| 9.4 | 6 | 57.8 | 37 | 32.8 | 21 | Pretest-posttest in web-based sessions enhance learning process |
| 18.8 | 12 | 50 | 32 | 31.3 | 20 | Images communicate information |
| 0 | 0 | 71.9 | 46 | 28 | 18 | Illustration and graphic quality are clear |
| 0 | 0 | 67.2 | 43 | 32.8 | 21 | Illustration and graphic quality are good |
| 3.1 | 2 | 65.6 | 42 | 31.3 | 20 | Illustration and graphic transmission are smooth |
| 0 | 0 | 67.2 | 43 | 32.8 | 21 | Illustration and graphic transmission do not lag |
| **Usability (24 marks) ±S.D (19.04±2.3**) | | | | | | |
| 14.1 | 9 | 53.1 | 34 | 32.8 | 21 | It was simple to use this website |
| 7.8 | 5 | 60.9 | 39 | 31.3 | 20 | It was easy to find the information |
| 0 | 0 | 60.9 | 39 | 39.1 | 25 | The organization of information on the website is clear |
| 3.1 | 2 | 62.5 | 40 | 32.8 | 21 | The navigation is clear |
| 7.8 | 5 | 64.1 | 41 | 28 | 18 | The navigation is easily understood |
| 0 | 0 | 54.7 | 35 | 45.5 | 29 | I am satisfied with this website |
| **Total score (116) ±S.D (96.39±4.2)** | | | | | | |
